# Injecting the Male Bladder without the Aid of a Catheter, and Some of Its Advantages

**Published:** 1876-11

**Authors:** 


### INJECTING THE MALE BLADDER WITHOUT THE AID OF A CATHETER, AND SOME OF ITS ADVANTAGES.

Dr. Hunter McGuire, Professor of Surgery in the Medical College of Virginia, contributes to the *Virginia Medical Monthly* (July, 1876) an interesting paper on this subject, from which the following is extracted:

About the time that Dr. Zeissl,* of Vienna, published his method of introducing fluids into the male bladder by means of an irrigator, I had under my care a case of vascular tumour of the bladder, in which the introduction of the catheter gave rise to severe chills and troublesome bleeding. This patient had local lancinating pains about the region of the bladder, hæmaturia, very frequent and painful micturition, and all the other indications of obstructive disease of the urinary passages. The use of the soft gum catheter, either to relieve the occasional retention of urine, or for the purpose of injecting the bladder, was always followed by chills and urethral fever, and sometimes by a profuse and sudden loss of blood, which blanched and exhausted the patient.

Instead of using Zeissl's method, described by Dr. Rose, of New York,† I resorted to the following simple means, which succeeded admirably: I took the common rubber bag syringe, holding about six ounces, provided with a stopcock and a gutta-percha nozzle tapering to a fine point. The syringe is one ordinarily used to inject the bladder through a catheter, and the nozzle tapers to a point, so that it may fit any catheter. This bag is filled with warm water, care being taken to exclude all of the air, and the nozzle oiled and introduced into the urethra for

---

* Wiener Med. Wochenschrift, 1874, Nos. 51 and 52.

†New York Medical Record, May, 1875, p. 330.

an inch or an inch and a half. With the forefinger and thumb of the left hand, the urethra is gently compressed around the nozzle of the syringe, the stopcock turned on so that the water may flow from the syringe into the urethra, while moderate and continued pressure is applied to the bag, and the fluid forced along the urethra into the cavity of the bladder. Care should be taken not to press the urethra too forcibly against the gutta-percha nozzle of the syringe ; gentle pressure only is necessary to prevent regurgitation of the fluid, and any rough manipulation will bruise the delicate mucous membrane of the urethra. The pressure applied to the bag should be gradual and continued, the surgeon making up his mind, the first time he injects the bladder in this way, to spend a few minutes in the operation ; but after both he and the patient have practiced it a few times it can be safely done more rapidly. The patient, to whose case I have referred, was in the habit of washing out his bladder twice a day by the plan described ; and he did it for himself, after a little instruction, so skillfully that he could empty the syringeful of water through the urethra into the bladder as rapidly as he could have done through an ordinary catheter. In making the injection, it is better to keep the penis parallel with the anterior abdominal wall, or pulled gently straight out from the pubis and perpendicular to that bone, so as, in the first instance, to give the urethra a single gentle curve ; or, in the second case, to make it an almost straight tube. Care should be taken not to stretch the penis too much while the injection is being made ; if stretched and pulled too much, the elastic urethral tube will be closed.

All of the fluid in front of the sphincter of the bladder is ejected with some little force by the urethra as soon as the nozzle of the syringe is taken away and the forefinger and thumb removed from the urethra ; and it is better to make some provision to catch this fluid and keep it from the patient's clothes. The quantity varies from one to two drachms to half an ounce or more, according to the size of the urethra, which, measured in this way, I have found to differ much, not only according to the age of the patient, but also in different adult individuals. Sometimes, when the injection has been completed, just before withdrawing the nozzle and while it is being taken away, I remove the pressure which has been kept up upon the bag, and the syringe will suck up most of the water left in the urethra. The injection can be made while the patient is in almost any position. I have used it while the man was standing up, or sitting, or lying down, and when he was tied and in the position to be cut for stone. The recumbent position is, however, the best. The fluid used should be warm; if cold, it is difficult to inject, and painful.

Unless there is some more serious objection than I have yet been able to discover to the use of injections of the bladder, performed in the way just described, I am sure this will prove a valuable addition to our present means of treating vesical disorders.

In the case mentioned of malignant vascular tumour of the bladder, where the soft gum catheter gave rise to serious bleeding and to severe urethral fever, nothing gave the patient so much comfort as the use of this gum bag syringe. He not only employed it morning and night to wash out the blood, mucus and pus which collected there, but sometimes as an injection of simple warm water stopped the pain and vesical tenesmus better than anything else.

Several times the bleeding was stopped by adding alum to the warm water, and occasionally borax and glycerin, or carbolic acid, was used. The mucous membrane of the urethra is much less sensitive to the influence of these and similar agents than the mucous membrane of the bladder, and no fear of injury to the urethra need deter the surgeon from injecting the bladder in this way. Two grains of sulphate of zinc to the ounce of rose-water is a mild injection for the urethra, but that strength of this drug would not be borne with impunity by the bladder.

